# Anti-interleukin-5 efficacy in an inclisiran-triggered eosinophilic myocarditis: a case report

**DOI:** 10.1093/ehjcr/ytaf127

**Published:** 2025-03-21

**Authors:** Francesco Tartaglia, Maria Rita Messina, Stefania Rizzo, Enrico Heffler, Cristina Panico

**Affiliations:** Department of Biomedical Sciences, Humanitas University, Via Rita Levi Montalcini, 4, 20079 Pieve Emanuele-Milan, Italy; Cardiovascular Department, IRCCS Humanitas Research Hospital, Via Alessandro Manzoni, 56, 20089 Rozzano-Milan, Italy; Department of Biomedical Sciences, Humanitas University, Via Rita Levi Montalcini, 4, 20079 Pieve Emanuele-Milan, Italy; Personalized Medicine, Asthma and Allergy Department, IRCCS Humanitas Research Hospital, Rozzano, Italy; Department of Cardiac, Thoracic, Vascular Sciences and Public Health, University of Padua, Via VIII Febbraio, 2, 35122 Padua, Italy; Department of Biomedical Sciences, Humanitas University, Via Rita Levi Montalcini, 4, 20079 Pieve Emanuele-Milan, Italy; Personalized Medicine, Asthma and Allergy Department, IRCCS Humanitas Research Hospital, Rozzano, Italy; Department of Biomedical Sciences, Humanitas University, Via Rita Levi Montalcini, 4, 20079 Pieve Emanuele-Milan, Italy; Cardiovascular Department, IRCCS Humanitas Research Hospital, Via Alessandro Manzoni, 56, 20089 Rozzano-Milan, Italy

**Keywords:** Eosinophilic myocarditis, Cardiac magnetic resonance, Interleukin-5, Mepolizumab, Multidisciplinary, Case report

## Abstract

**Background:**

Eosinophilic myocarditis is a rare condition that can be associated with hypersensitivity reactions. Endomyocardial biopsy (EMB) is required for diagnosis, especially when cardiac magnetic resonance (CMR) is inconclusive. Immunosuppressive treatment is usually limited to corticosteroids.

**Case summary:**

Two days following an inclisiran injection for dyslipidaemia management, a 72-year-old Caucasian male with a history of coronary artery disease experienced progressive shortness of breath. Suspecting a hypersensitivity reaction to inclisiran, corticosteroids were administered. After discontinuing corticosteroids, the patient experienced recurrent dyspnoea. Laboratory tests indicated eosinophilia, increased serum immunoglobulin E (IgE), and positive specific serum IgE for *Aspergillus fumigatus*. Imaging tests and a lung biopsy revealed pulmonary aspergillosis, while CMR showed myocardial inflammation. The patient was initially treated with itraconazole and steroid therapy. However, he was re-hospitalized for worsening of the cardiac and respiratory condition after tapering steroids. A diagnosis of severe eosinophilic asthma associated with allergic bronchopulmonary aspergillosis was established. Löffler’s endocarditis related to *Aspergillus*-induced eosinophilia was suspected and confirmed by repeated CMR and an EMB. Mepolizumab, an interleukin-5 (IL-5) inhibitor, was initiated resulting in symptom resolution and absence of inflammation at the 6-month follow-up CMR.

**Discussion:**

This case describes an uncommon response to inclisiran that resulted in *Aspergillus*-induced, steroid-dependent, eosinophilic systemic inflammation, resulting in severe asthma and endocarditis. It demonstrates how biological IL-5 inhibitors are effective in treating both components, emphasizing the necessity of a multidisciplinary strategy.

**Learning points**

- To highlight the necessity of endomyocardial biopsy when eosinophilic myocarditis is suspected
- To demonstrate the usefulness of a multidisciplinary approach when multiple organ involvement is present
- To show the efficacy of interleukin-5 inhibitors in cases of steroid-resistant eosinophilic myocarditis

## Introduction

Eosinophilic myocarditis (EM, also called *Löffler’s endocarditis*) is a rare cardiac disorder caused by eosinophilic infiltration with variable clinical presentations. It has been associated with hypersensitivity reactions, immune disorders, hypereosinophilic syndrome (HES), and cancer, but in most cases, the cause remains undetermined.^1,2^

Cardiac magnetic resonance (CMR) can provide a diagnosis, but in certain circumstances, non-specific results may require endomyocardial biopsy (EMB).^3,4^ The cornerstone of treatment is immunosuppression, which is often restricted to corticosteroid therapy.

We report an unusual case in which Löffler’s endocarditis and severe eosinophilic asthma associated with allergic bronchopulmonary aspergillosis (ABPA) arose after inclisiran administration. Treatment with an anti-interleukin-5 (IL-5) monoclonal antibody (mepolizumab) resulted in improvement of both conditions. The timeline of events is summarized in *Table 1*.

**Table 1:** Timeline of relevant events

Timeline	Events	Examinations
Day 0	First inclisiran injection	
Day 2	Allergic reaction → oral corticosteroids	-Eosinophilia
Third month	Recurrent dyspnoea after steroid interruption	-CT: lymphadenopathies, subpleural thickening
Fourth month		-Lung biopsy: *Aspergillus fumigatus* -CMR: subacute inflammatory heart injury
Fifth month	Diagnosis: ABPA → prednisone, ICS/LABA	
10th month	AHF after steroid interruption	-Eosinophilia -CMR: increased inflammation -EMB: eosinophilic myocarditis
11th month	Mepolizumab started	
17th month	Asymptomatic	-CMR: absence of active inflammation

ABPA, allergic bronchopulmonary aspergillosis; AHF, acute heart failure; CMR, cardiac magnetic resonance; CT, computed tomography; EMB, endomyocardial biopsy; ICS, inhaled corticosteroids; LABA, long-acting beta2-agonists.

## Summary figure

CMR, cardiac magnetic resonance; EBM, endomyocardial biopsy; HF, heart failure; PET, positron emission tomography.

**Figure:**
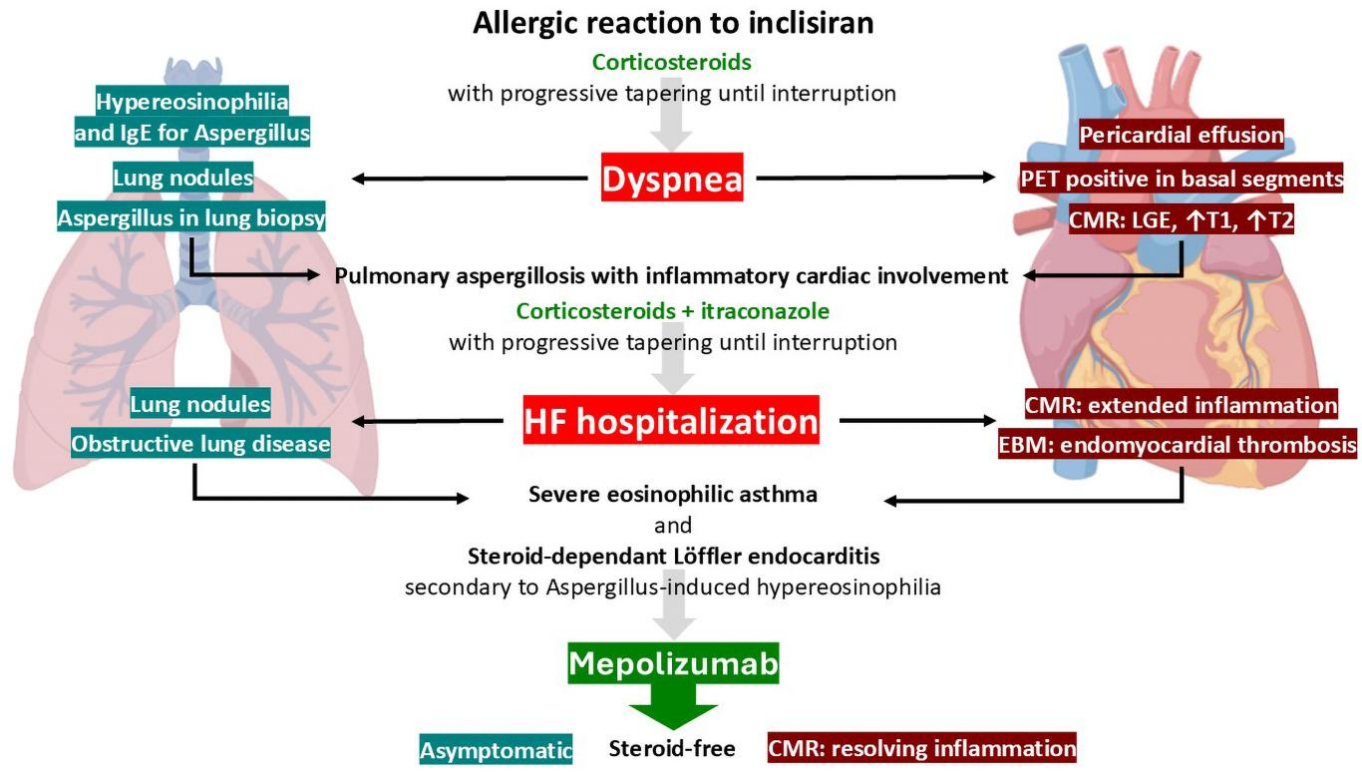


## Case presentation

A 72-year-old Caucasian male presented to our outpatient cardiology clinic. His medical history included smoking, arterial hypertension, dyslipidaemia, ischaemic stroke, and chronic coronary syndrome. He had no history of allergies or risk factors for immunodeficiency. His current medications included rosuvastatin 40 mg plus ezetimibe 10 mg per day. Given his LDL cholesterol level of 91 mg/dL, a proprotein convertase subtilisin/kexin type 9 inhibitor medication was suggested. After accepting, the patient had his first injection of inclisiran.

Two days later, the patient presented to our emergency department with complaints of dyspnoea. Examination revealed diffuse wheezing, and chest X-ray showed mild bronchial wall thickening. Eosinophilia was detected (up to 630 cells/mm^3^). Respiratory insufficiency was diagnosed, and on suspicion of inclisiran as a potential trigger, the drug was discontinued, intravenous corticosteroid therapy was administered, and the patient was discharged with a tapering regime of oral prednisone.

**Figure 1:**
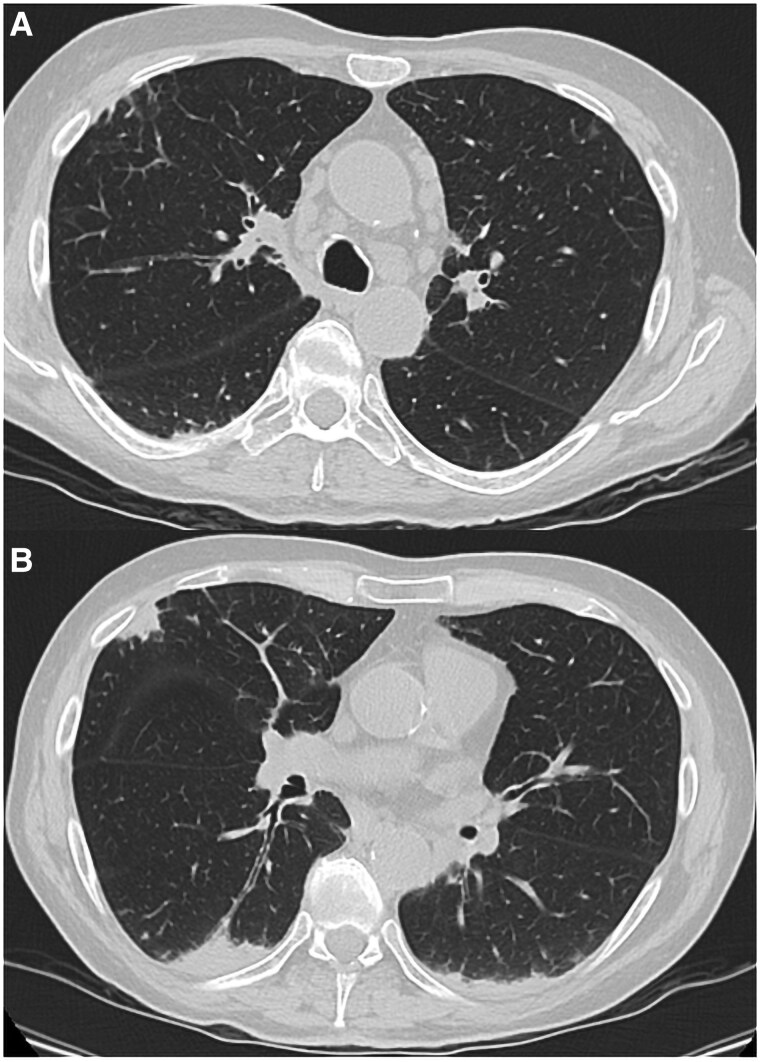
Computed tomography scan showing numerous enlarged mediastinal lymph nodes (*A*) and subpleural thickening of the posterior and medium right lobes and the posterior left lobe (*B*).

Three months later, just 1 week after ceasing oral corticosteroids, recurrent dyspnoea and a dry cough reappeared. Blood exams showed eosinophilia (up to 880 cells/mm^3^) and increased serum immunoglobulin E (IgE) levels (1964 international units (UI)/mL, reference value < 85 UI/mL). Parasitic infection was ruled out through faecal parasite test and parasites serologies (immunoglobulins M and G for amoebiasis and echinococcosis). Serum-specific IgE for *A. fumigatus* was assessed and resulted mildly positive (0.23 kilounits/L, reference value < 0.1 kilounits/L). Computed tomography (CT) scan revealed several mediastinal lymphadenopathies, together with bilateral areas of subpleural thickening and a 6 mm flap of pericardial effusion (*Figure 1*). A positron emission tomography (PET) scan was therefore performed, confirming the presence of multiple hypermetabolic nodules in lung parenchyma and lymph nodes. Additionally, it revealed a notable accumulation of the trace in the basal segments of the heart. Lung function tests revealed reversible bronchial obstruction [forced expiratory volume (FEV1): 56% of the predicted value; FEV1/forced vital capacity (FVC): 63%; FEV1 improvement of 18% corresponding to 300 mL after bronchodilation test]. A lung biopsy was obtained detecting *A. fumigatus*.

**Figure 2:**
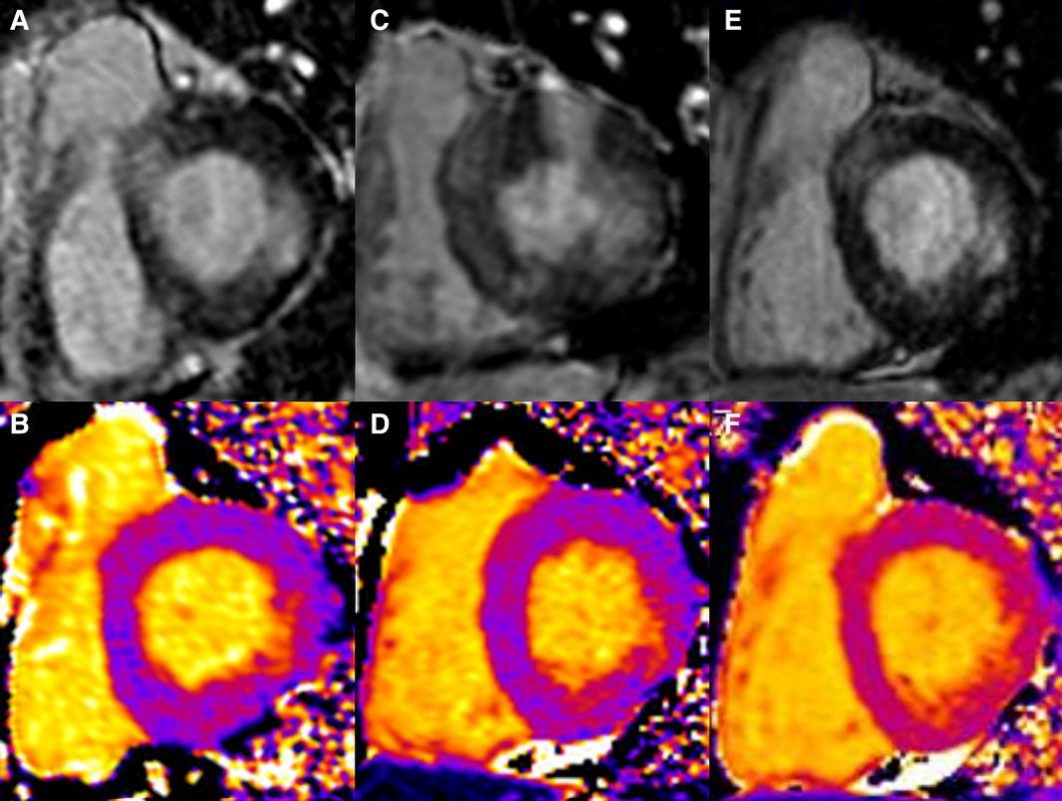
The findings from the three cardiac magnetic resonances. (*A*, *C*, and *E*) Late gadolinium enhancement of first, second, and third cardiac magnetic resonance, respectively. (*B*, *D*, and *F*) Corresponding T1 relaxation times.

Meanwhile, a full cardiac evaluation was performed. The electrocardiography and echocardiogram were unremarkable, and no rise in cardiac enzymes was detected. On the other hand, a CMR revealed increased T1 and T2 relaxation time (1090 and 54 ms, respectively) in the basal lateral wall. Late gadolinium enhancement (LGE) was detected in the same area, confirming the presence of a subacute inflammatory heart injury (*Figure 2A* and *B*).

Given the clinical presentation and diagnostic findings, the diagnosis of asthma with associated ABPA with inflammatory cardiac involvement was postulated. Itraconazole and oral prednisone were thus prescribed, and proper treatment with inhaled corticosteroids/long-acting beta2-agonists combination was started.

At 3-month follow-up, the patient was asymptomatic. Blood tests revealed no eosinophilia, and PET and CT scan revealed the complete regression of anatomical and metabolic anomalies. Prednisone was discontinued.

Approximately 15 days later, the patient was hospitalized for acute congestive heart failure. A marked increase in serum IgE levels (3047 UI/mL) was observed, and a CT scan revealed new pulmonary consolidations that recalled earlier-identified ones. The brain natriuretic peptide level was 810 pg/mL. Cardiac magnetic resonance was repeated. Systolic function was moderately reduced, and T1 relaxation time was found to be increased both globally (1040 ms) and locally in the basal postero-lateral wall (1150 ms), as well as T2 (55 ms). Late gadolinium enhancement was detected in the previously identified area, as well as in the basal anterior and inferior regions (*Figure 2C* and *D*).

**Figure 3:**
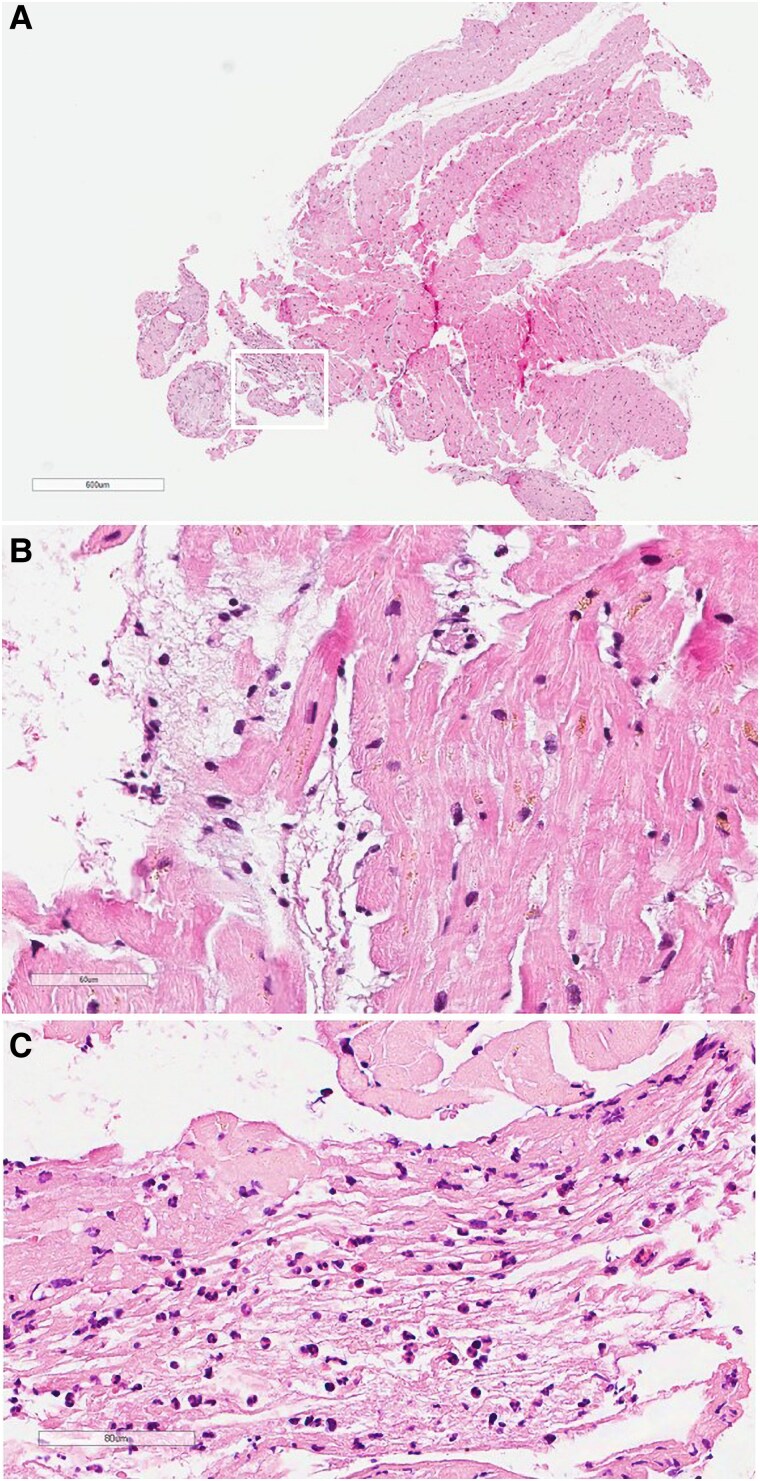
Endomyocardial biopsy findings. (*A*) Panoramic view of an endomyocardial biopsy sample. (*B*) At higher magnification, note eosinophilic infiltration of the myocardium. (*C*) Mural thrombus rich in eosinophils is also present, findings in keeping with Löffler’s endocarditis [haematoxylin–eosin stain: (*A*) bar 600 micron, (*B*) 60 micron, and (*C*) 80 micron)].

Left ventricular EMB of multiple myocardial areas was performed, with most of the samples obtained from the basal walls as suggested by LGE distribution. The biopsy showed focal and interstitial fibrosis, eosinophilic cells, and a fragment with endomyocardial thrombosis (*Figure 3*). Löffler’s endocarditis secondary to *Aspergillus*-induced hypereosinophilia was eventually diagnosed.

Concomitantly, the patient again developed frequent dyspnoea and cough, despite an increase in treatment level (beclometasone/formoterol/glycopyrronium inhaled combination). A diagnosis of severe eosinophilic, corticosteroid-dependent asthma was made. A multidisciplinary team composed of cardiologists, allergologists, and pulmonologists evaluated the patient for a potential biological treatment approach. Given the diagnosis of severe eosinophilic asthma, mepolizumab (an anti-IL-5 monoclonal antibody) was initiated at a dose of 300 mg every 4 weeks.

Six months later, the patient was asymptomatic from both respiratory and cardiological points of view, and the treatment was well tolerated with no reported adverse effects. Asthma was under proper control, and no exacerbation was reported. A follow-up CMR showed absence of active inflammation, with normal T2 time both globally and on the lateral wall (49 ms). T1 remained enhanced in the area where LGE was present (1128 ms), but no new areas of LGE were detected (*Figure 2E* and *F*). Given the excellent response and the high risk of exacerbations, cyclic injections of mepolizumab were continued, while oral prednisone was not reintroduced.

## Discussion

Eosinophilic myocarditis is a rare, complex disease whose management is still not addressed in evidence-based guidelines. It should be suspected when signs and symptoms of hypersensitivity (such as wheezing and eosinophilia) are combined with cardiac findings (such as altered electrocardiogram, elevated troponin, and structural and functional myocardial anomalies). Cardiac magnetic resonance may be inconclusive, as in the presented case. Therefore, EMB should be performed whenever possible in ambiguous cases, as recommended by the 2023 ESC guidelines for the management of cardiomyopathies.^5^

Treatment options are usually limited to corticosteroids. Mepolizumab, targeting circulating IL-5, prevents the binding with its receptor on eosinophils and their progenitors and is currently approved by the European Medical Agency for severe eosinophilic asthma, chronic rhinosinusitis with nasal polyps, eosinophilic granulomatosis with polyangiitis, and HES, but its use in EM is limited to few reported cases.^6–8^

Our case has several learning points. First, it emphasizes the importance of careful monitoring for adverse reactions. Although it remains uncertain if ABPA was primarily triggered by the adverse drug reaction or by the use of steroids, the injection of Inclisiran was the *primum movens* of the events. Second, it stresses the investigation of potential cardiac involvement in cases of eosinophilia and highlights the limitations of multimodality imaging and the necessity of EMB when EM is suspected. The site of the biopsy should be selected according to the CMR results, especially if a patchy involvement of the myocardium is suspected. Third, it underlines that ABPA can be diagnosed even without a previous history of respiratory disease, although uncommon. Lastly, it shows the efficacy of biological treatment in cases of steroid-resistant EM in terms of symptom resolution and inflammation control and the benefit of a multidisciplinary approach when multiple organ involvement can be managed simultaneously.

## Data Availability

Non-identifiable data underlying this article will be made available upon reasonable request to the corresponding author.

## Lead author biography

Dr Francesco Tartaglia earned his MD from Sapienza University of Rome with a thesis on lung–heart interactions in pneumonia. He is currently a resident in cardiology at Humanitas Research Hospital in Milan. His interests range from clinical cardiology to advanced imaging and interventional cardiology.
